# Evaluation of the orientation dependence of optically stimulated luminescence dosimeters in computed tomography dosimetry using an anthropomorphic phantom under clinical imaging conditions

**DOI:** 10.20407/fmj.2025-014

**Published:** 2025-11-05

**Authors:** Takuma Ichikawa, Yusei Nishihara, Tomonobu Haba, Yasuki Asada

**Affiliations:** 1 Major in Medical Science, Graduate School of Health Science, Fujita Health University, Toyoake, Aichi, Japan; 2 Department of Radiology, Fujita Health University Okazaki Medical Center, Okazaki, Aichi, Japan; 3 Department of Radiology, Fujita Health University Hospital, Toyoake, Aichi, Japan; 4 Department of Medical Imaging Technology, School of Medical Science, Fujita Health University, Toyoake, Aichi, Japan; 5 Department of Diagnostic Instrumentation Engineering, School of Medical Science, Fujita Health University, Toyoake, Aichi, Japan

**Keywords:** Optically stimulated luminescence dosimeters, Computed tomography, Dosimetry

## Abstract

**Objective:**

Small dosimeters such as optically stimulated luminescence dosimeters (OSLDs) and thermoluminescence dosimeters (TLDs) are used in anthropomorphic phantoms to measure actual organ exposure in X-ray computed tomography (CT) examinations. Nagase-Landauer currently supplies nanoDot dosimeters that employ OSLDs. The flat structure of nanoDot dosimeters causes their sensitivity to fluctuate according to the direction of the incident X-rays. We evaluated the effect of OSLD angle on measured values when dosimeters were placed within an anthropomorphic phantom and dosimetry was performed to mimic X-ray CT examinations.

**Methods:**

The dependence of OSLD placement angle on measured values was evaluated at eight points in the thorax and abdomen of the anthropomorphic phantom including the lung field, near the center of the phantom, near the body surface, and near the tissue boundary.

**Results:**

The system error for OSLDs positioned at different angles in the anthropomorphic phantom was 6.58%. Variation of measured values with the OSLD placement angles in the phantom resulted in errors ranging from –7.3% to +5.4%.

**Conclusion:**

Placement angle had little effect on measurement values when system error was considered. Therefore, it is not necessary to consider placement angle when measuring organ doses using OSLDs.

## Introduction

The International Commission on Radiological Protection (ICRP) states that patient exposure dose in X-ray computed tomography (CT) examinations is not negligible and mentions the importance of optimization and dose assessment.^1^ In CT examinations, it is necessary to display dose indices such as the CT dose index volume (CTDIvol) and dose length product (DLP), as specified in the 2002 International Electrotechnical Commission recommendations (60601-2-44).^2^ These dose indices need to be displayed on the CT console, where they can easily be evaluated. However, CTDIvol and DLP are dose indices and cannot be used to evaluate exposure dose to the patient. Based on the ICRP recommendations,^3^ equivalent dose and effective dose are used to assess patient exposure dose. These parameters can be calculated by Monte Carlo simulation or measured using an anthropomorphic phantom.^4–7^

In actual measurements, small dosimeters such as optically stimulated luminescence dosimeters (OSLDs) and thermoluminescent dosimeters (TLDs) are placed in an anthropomorphic phantom. The equivalent dose for each organ is measured and the effective dose is calculated. The calculation formulas for the equivalent dose and effective dose are as follows.^8^

Equivalent Dose

(1.1) $$H_{T}=\sum_{R}\omega_{R}\cdot D_{T,R}$$

H_Τ_: Equivalent dose in tissue or organ T (in sieverts, Sv)

D_T,R_: Absorbed dose in tissue T from radiation type R (in grays, Gy)

ωR: Radiation weighting factor for radiation type R

R: Type of radiation (e.g., X-rays, gamma rays, alpha particles, neutrons)

Effective Dose

(1.2) $$E=\sum_{T}\omega_{T}\cdot H_{T}=\sum_{T}\omega_{T}\cdot \sum_{R}\omega_{R}\cdot D_{T,R}$$

E: Effective dose (in sieverts, Sv)

H_Τ_: Equivalent dose in tissue or organ Τ

ω_Τ_: Tissue weighting factor for tissue or organ Τ

Τ: Tissue or organ

ωR: Radiation weighting factor

D_Τ, R_: Absorbed dose in tissue Τ from radiation type R

Compared with TLDs, OSLDs are less expensive and have almost no fading effect.^9^ The nanoDot dosimeter (Landauer, Glenwood, IL) is a very small OSLD (10×10×2 mm) in current use.^10–12^ The anthropomorphic phantom manufactured by Computerized Imaging Reference Systems (CIRS; Norfolk, VA) is compatible with the nanoDot dosimeter.^13^

The nanoDot dosimeter is a flat OSLD and its sensitivity has been confirmed to vary with the direction of the incident X-rays.^14^ Previous studies have evaluated the angular dependence of OSLDs placed in air and under the high-energy conditions used in radiation therapy.^15,16^ In addition, the angular dependence within a CTDI phantom has been assessed.^14^ However, the angular dependence of OSLD placement has not yet been investigated in an anthropomorphic phantom containing various materials with organ-equivalent density under diagnostic X-ray energy conditions. In this study, the directional dependence caused by the angle of OSLD placement in measurements using an anthropomorphic phantom is evaluated with the aim of ensuring accurate dose assessment in the human body.

## Materials and Methods

Ethical approval was not required because this study was conducted using a phantom model. Measurements were obtained using an X-ray CT scanner (Aquilion ONE NATURE Edition, Canon Medical Systems, Otawara, Japan), nanoDot OSLDs (Landauer), a microSTAROSLD reader (Landauer), and an ATOM anthropomorphic phantom (Model 70, CIRS).

Each OSLD was composed of Al_2_O_3_:C with a diameter of 4 mm and thickness of 0.3 mm. This disk was enclosed in a plastic case measuring 10×10×2 mm. The OSLDs were capable of measurements over a wide energy range from 5 keV to 20 MeV. OSLDs exhibit linear response characteristics even in the general diagnostic X-ray range (30–150 kVp).^14^ In this study, up to 80 OSLDs were used.

The ATOM anthropomorphic phantom (Model 70) simulated an average adult male with a height of 175 cm and weight of 70 kg. The phantom consisted of tissue-equivalent materials that mimic the radiation absorption properties of various tissues such as skin, fat, muscle, bone, lungs, and organs.^13^

### Evaluation of Signal Fading Characteristics of OSLDs with Repeated Readouts

**Figure 1:**
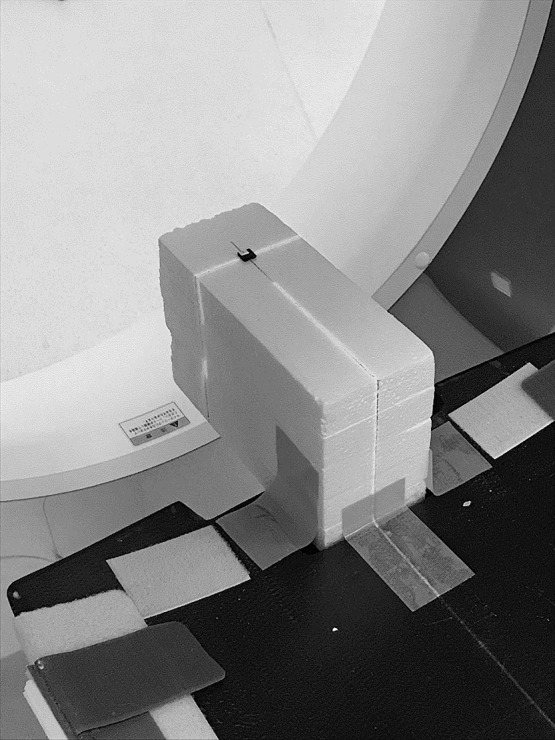
Photograph of OSLD placement during OSLD system characterization.

Although OSLDs can take multiple readings, it is known that the counts obtained decay with each reading.^8^ Therefore, we first evaluated this attenuation characteristic. One OSLD was fixed to the isocenter of the CT scanner and irradiated by conventional X-ray CT scanning using a tube voltage of 120 kVp, tube current of 200 mA, rotation speed of 1 s/rotation, L bowtie filter, beam width of 0.5×80 mm, and orbital synchronization (Figure 1). Twenty-six different OSLDs were read 20 times each and their attenuation characteristics were evaluated. To assess the system error of the OSLD measurements, 20 OSLDs were irradiated with X-rays under identical conditions, and each OSLD was measured three times. The signal in each OSLD was retained within an electron trap. Because the signal decreases exponentially, signal attenuation was evaluated using an exponential approximation.

### Evaluation of OSLD orientation dependence

**Figure 2:**
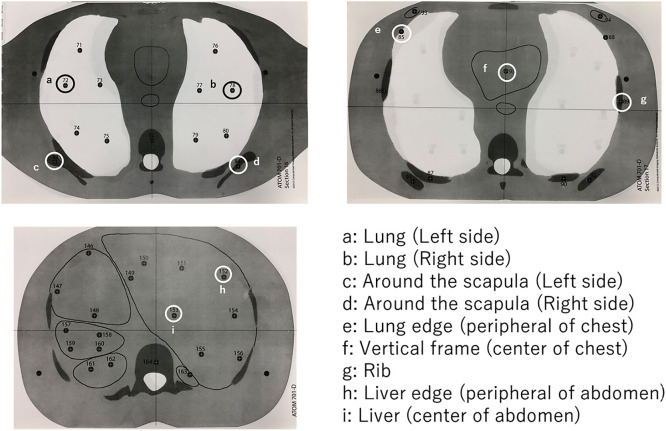
CT images showing OSLD insertion locations in the human phantom.

The dependence of OSLD orientation on count was evaluated at eight points in the anthropomorphic phantom (Figure 2) to cover the regions of the lung fields, near the center of the phantom, near the body surface, and near tissue boundaries. The placement angle of the dosimeter was defined as 0° when it was horizontal to the bed. Measurements were taken at intervals of 30° up to 150°. This angulation was applied along the *x*=*y* axis on the axial plane, where the horizontal direction is defined as the *x*-axis and the vertical direction as the *y*-axis. Each measurement point was evaluated once for each angulation, and each OSLD was read five times. Fifty-four OSLDs were used in this experiment. Measurement errors caused by changes in OSLD placement angles in each organ were evaluated for statistical significance with reference to the system error of the OSLD in accordance with the Guide to the Expression of Uncertainty in Measurement (GUM) framework.^17^ The corresponding standard uncertainty was calculated using the following equation:

(2.1) $$u\left(x_{i}\right)=\frac{S.E.}{\sqrt{3}}$$

$u\left(x_{i}\right)$: standard uncertainty

S.E.: system error

The combined standard uncertainty can be derived using the law of propagation of uncertainty, which can be expressed by:

(2.2) $$u_{e}(\Delta )=\sqrt{{u\left(x_{i}\right)}^{2}+{u\left(x_{i}\right)}^{2}}$$

$u_{e}(\Delta )$: combined standard

To evaluate the uncertainty at a 95% confidence level, a coverage factor of 2 was applied. The expanded uncertainty was calculated as follows:

(2.3) $$U=2\times u_{e}(\Delta )$$

U: expanded uncertainty

The measurement error was compared with the expanded uncertainty to determine the presence or absence of statistical significance.

The exposure conditions for helical scanning were a tube voltage of 120 kVp, tube current of 300 mA, rotation speed of 0.5 s/rotation, L bowtie filter, beam width of 0.5×80 mm, helical pitch of 65, and scan range from the chest to the pelvis. Orbit synchronization was turned on at the time of measurement to ensure that the start angle of the helical scan would be the same for each acquisition.

## Results

### Evaluation of Signal Fading Characteristics of OSLDs with Repeated Readouts

**Figure 3:**
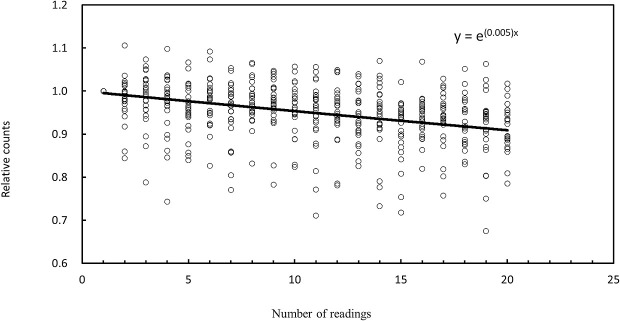
Relationship between counts and the number of readings during OSLD system characterization.

Figure 3 shows decay curves for each of the 26 OSLDs relative to the first reading. A single reading resulted in a count decay of approximately 0.5%. The measurement error of the system was 6.58%.

### Evaluation of OSLD orientation dependence

**Figure 4:**
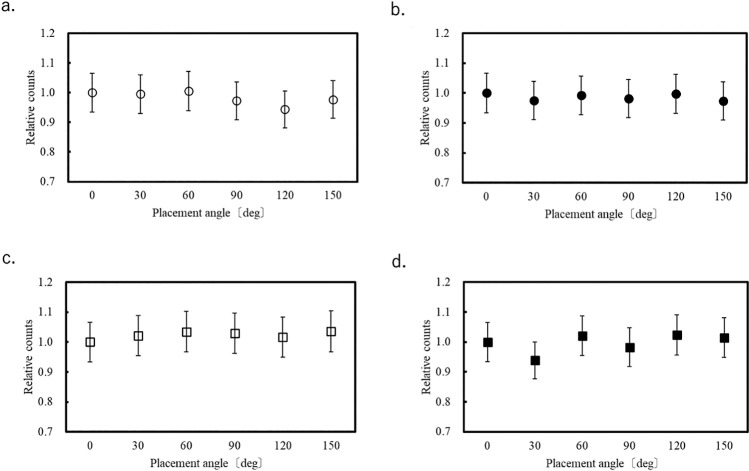
Count fluctuation depending on OSLD placement angle in Lung 1.

**Figure 5:**
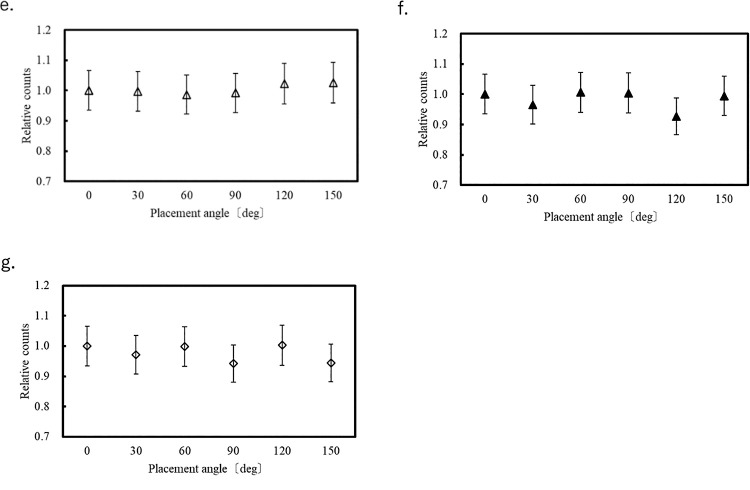
Count fluctuation depending on OSLD placement angle in Lung 2.

**Figure 6:**
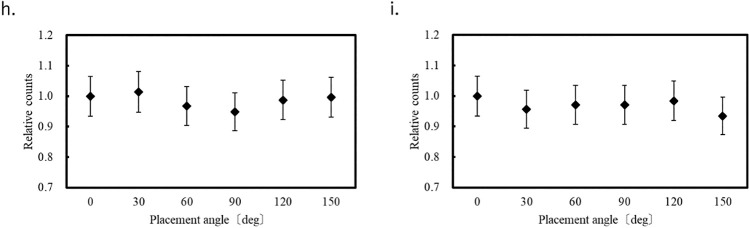
Count fluctuation depending on OSLD placement angle in the abdomen.

Figure 4–6 show the variation of the count at each of the eight measurement points for each OSLD placement angle relative to that at 0°. Error bars correspond to the system error (6.58%) evaluated for the OSLDs. Because the OSLD system used in this study exhibits 0.5% signal attenuation with each readout, a correction based on the number of readouts was applied using the following equation.

(3.1) $${RV}_{a}=\frac{{RV}_{b}}{R_{n}e^{0.5}}$$

RVa: Adjusted readout value

RVb: Readout value before correction

Rn: Number of readouts

At each of the eight measurement points in the phantom, the measured values at different angular orientations relative to 0° varied within the range of –7.3% to +5.4%.

Considering that the system error of the OSLD was 6.58%, the corresponding standard uncertainty was evaluated using the equations (2.1–2.3) presented in the Materials and Methods section, in accordance with the GUM framework.

$$u\left(x_{i}\right)=\frac{6.58}{\sqrt{3}}=3.80\%$$

$$u_{e}(\Delta )=\sqrt{(3.80{)}^{2}+(3.80{)}^{2}}=5.37\%$$

U=2×5.37=10.7%

In the present study, the measurement errors ranged from –7.3% to 5.4%. However, both –7.3%±10.7% and 5.4%±10.7% include 0% within their 95% confidence intervals, indicating that no statistically significant difference was observed at the 95% confidence level.^17^

## Discussion

The system error of the evaluated OSLD system was 6.58% and errors of –7.3% to 5.4% occurred at the eight measurement points in the phantom. Because the interelement variability of OSLDs has been reported to range from 5% to 10%, the system error of 6.58% observed in this study is acceptable.^9,10^ Considering that the system error of the OSLD was 6.58%, the evaluation based on the GUM framework indicated that no statistically significant differences were observed at the 95% confidence level.^17^

Therefore, compared with the system error of the OSLD, the measurement errors resulting from changes in the OSLD placement angle were small at most measurement points. Therefore, the influence of placement angle on count is minimal. In the center of the chest (position f in Figure 2), we measured an error that exceeded the OSLD system error of 6.58%. It is unclear why the placement angle affected the measured value at this location because it occurred at the center of the phantom and there were no large tissue changes around the OSLD. One possible cause is the large fluctuation in measurements during OSLD reading, as shown in Figure 3.

A previous study examined the orientation dependence of OSLDs used in general radiography, mammography, and CT.^14^ Among these, the CT results showed the least apparent variation (a maximum decrease of 10% in the *z*-direction) for the reason that in addition to X-rays being directed toward the center of the bore from directions of 360°, scattered X-rays generated by the polymethylmethacrylate (PMMA) material of the computed tomography dose index (CTDI) phantom also strongly affect the measured values, and the angle at which OSLDs are placed in the phantom is relatively insignificant in comparison.^14^ In the evaluation of orientation dependence in CT, modifications were made to a standard CTDI phantom by drilling holes to allow insertion of dosimeters. Using a CTDI phantom with a diameter of 32 cm representing the torso, measurements were performed at depths of 0 cm (i.e., the phantom surface) and 1, 9, 12, and 16 cm. The assessment was conducted by varying the placement angle of the OSLDs under a beam width of 40 cm and tube voltage of 80 or 120 kVp.

Compared with that in a CTDI phantom, verification in an anthropomorphic phantom is more complex because it contains materials with various densities. PMMA has a uniform density of about 1.19 g/cm^3^. In contrast, an anthropomorphic phantom has a density of 0.28 g/cm^3^ in the lung, which is a target organ and the lowest density in the phantom; 1.05 g/cm^3^ in soft tissue; and 1.92 g/cm^3^ in the scapular cortex.^18^ We expected that the effect of OSLD placement angle on the measured values would be greatest in the lung and bone, which have quite different densities to that of PMMA; however, no significant effect was found. In the lung, which has low density, fewer scattered X-rays were generated in the lung itself than in other organs, but scattered X-rays were generated by the surrounding medial wall and soft tissue near the body surface. The same was true for bone, which was affected by scattered X-rays generated not only in bone but also in the surrounding tissue. Therefore, it is considered that the measured values are not affected by the structure at the measurement point.

We found no difference in measured values between the center and edge of the phantom in terms of OSLD orientation. The majority of X-rays in a CT phantom are scattered X-rays from within the phantom itself. Therefore, there is relatively little measurement error caused by OSLD orientation direction.

It has been reported that orientation dependence is affected by the X-ray energy, and that the lower the X-ray energy, the greater the error of measurement values caused by OSLD orientation.^14^ Diagnostic CT uses relatively high-energy X-rays, but low-energy X-rays are sometimes absorbed in the phantom. Therefore, X-rays passing through the phantom have a higher effective energy than direct X-rays and show no significant change in spectral shape between the center of the phantom and near its surface.^19^ Thus, it is thought that the effect of OSLD orientation on the measured values is small. However, this study has several limitations. The results were obtained under fixed conditions with a tube voltage of 120 kV and a constant tube current. Furthermore, the evaluation was conducted with the target organ included within the imaging field; therefore, the effects outside the irradiation field were not assessed. Future investigations under different scanning conditions and organ positions are needed to confirm and extend these findings.

## Conclusions

The system error for OSLDs positioned with different orientations in an anthropomorphic phantom was 6.58%. Placement angle had little effect on measurement values when system error was considered. Therefore, it is not necessary to consider placement angle when measuring organ doses using OSLDs. Although no direct comparison with TLDs was conducted in this study, OSLDs displayed minimal angular dependence and offer practical advantages such as ease of use, suggesting their potential utility in organ dose measurements in CT.
